# Enterocin BacFL31 from a Safety* Enterococcus faecium* FL31: Natural Preservative Agent Used Alone and in Combination with Aqueous Peel Onion (*Allium cepa*) Extract in Ground Beef Meat Storage

**DOI:** 10.1155/2019/4094890

**Published:** 2019-04-18

**Authors:** Ahlem Chakchouk Mtibaa, Slim Smaoui, Hajer Ben Hlima, Imen Sellem, Karim Ennouri, Lotfi Mellouli

**Affiliations:** ^1^Laboratory of Microorganisms and Biomolecules, Center of Biotechnology of Sfax, Road of Sidi Mansour Km 6, P. O. Box 1177, 3018, University of Sfax-Tunisia, Tunisia; ^2^Algae Biotechnology Unit, Biological Engineering Department, National School of Engineers of Sfax, University of Sfax, Sfax 3038, Tunisia

## Abstract

Safety aspects and probiotic properties of* Enterococcus faecium *FL31 strain producing an enterocin, named BacFL31 were previously demonstrated. Taking into account its originality, the enterocin BacFL31 was added alone at 200 AU/g or in combination with the aqueous peel onion (*Allium cepa*) extract (APOE) at 1.56 ± 0.3 mg/mL to ground beef meat. Its biopreservative effect was evaluated by microbiological, physicochemical and sensory analyses during 14 days at 4°C. The APOE was characterized for its phytochemical content: total phenolic (TPC), flavonoids (TFC) and tannins contents (TAC), its antioxidant capacity using the* in vitro* 1,1-diphenyl-2-picrylhydrazyl (DPPH) and its antilisterial activity. APOE had a high TPC, TFC and TAC respectively with 140 ± 2.05 (mg GAE/g), 35 ± 0.5 (mg QE/g) and 20.6 ± 1.4 (mg CE/g). Equally, APOE showed a potential radical scavenging activity compared to the butylated hydroxytoluene (BHT), with an anti-radical power (ARP) of 46 ± 1.5. During 14 days of storage at 4°C, the combination between APOE and BacFL31 limited the microbial deterioration (*P* < 0.05), led to a decrease in thiobarbituric acid reactive substances (TBARS) values and slowed down the metmyoglobin (MetMb) and carbonyl group accumulation and delayed the disappearance of sulfphydryl proteins (*P* < 0.05). The combination was also efficient (*P* < 0.05) against microflora proliferation, decreased primary and secondary lipid oxidation (*P* < 0.05), reduced protein oxidation and enhanced significantly (*P* < 0.05) the sensory attributes. Thus, the enterocin BacFL31 use from a safe* Enterococcus faecium *combined with APOE as a potential natural preservative to biocontrol ground beef was promising as it was effective at low concentration. The data lay bases for new tests to be carried out in other food matrices.

## 1. Introduction

Due to its composition, meat and meat products are prone for growth of several microorganisms and pathogenic bacteria as well as oxidation reactions [1, 2]. These latter have been considered as one of the most significant causes of quality deterioration in meat and meat products during processing and storage [3–5]. The main targets of this type of redox reaction in meats are lipids and proteins. In this regard, lipid oxidation affects unsaturated lipids and leads to development of rancidity and degradation of sensory and nutritional value reducing their shelf-life time [6, 7]. In addition, during protein oxidation, reactive oxygen species may attack the side chain of amino acids and the peptide backbone, which leads to formation of carbonyl compounds, decrease in the sulfhydryl contents, loss of essential amino acids and water-holding capacity, reduction in protein solubility and eventually degradation of texture and color [8–10].

The use of additives with antioxidant properties and antimicrobial activities could be an adequate strategy to deal with the oxidation and the microbial proliferation in meat and meat products [11, 12]. However, consumer concerns about the relationship between health and nutrition, challenge food technologists to develop healthy meat products with improved characteristics. In order to answer the demand from consumers, many newly products with natural preservative have been developed in order to reduce the use of synthetic additives which have been linked to health risks is increasing.

Amongst others, the use of essentials oils, plant extracts or bacteriocins from lactic acid bacteria (LAB) constitute different ways to control lipid and protein oxidation and pathogenic bacteria proliferation in meat systems [3, 13–15]. In this context, natural antioxidants from plant extracts have been obtained from different sources such as fruits: grapes, pomegranate, date, kinnow, vegetables: broccoli, potato, drumstick, pumpkin, curry, nettle, herbs and spices, and investigated to decrease lipid oxidation and to preserve and improve the overall quality of meat and meat products [2, 11, 16].

Onions (*Allium cepa*) are utilized in various types of food, and they are one of the major sources of antioxidant content [17]. The major flavonoids found in onion dry peel, considered usually as waste, contain large amounts of phenolic compounds, such as quercetin, the major flavonoid, gallic acid, ferulic acid, and kaempferol which are effective antioxidants and have many pharmacological properties [18, 19]. The onion extracts had been widely studied on its antioxidant properties were largely evaluated in food preservation. For example, the brined onion extracts could enhance the quality of turkey breast rolls during seven days of refrigerated storage [20, 21]. Ground beef patties with added onion tissue showed decreased mutagenicity [22] and formation of heterocyclic aromatic amines during frying [23]. Equally, onion peel extract was demonstrated to be a very effective inhibitor of lipid oxidation and has potential as a natural antioxidant in raw ground pork [24].

On the other hand, bio-preservation by bacteriocins produced by LAB has gained increased attention as means of naturally controlling the safety and extending the shelf life of different meat matrix [15, 25]. The most common protective cultures belong to* Lactobacillus* and* Bifidobacterium* genera, while strains of* Enterococcus* spp. are occasionally used [26]. Most of these microorganisms are able to produce bacteriocins, named enterocins, active against pathogenic and spoilage bacteria. Therefore, enterocin produced by* Enterococcus* spp. are interesting candidates for guaranteeing the safety of meat and meat products [27, 28]. In this context, enterocins A and B have been extensively studied for their strong antibacterial properties especially in meat products [29]. Likewise, in our previous work, the addition of enterocin BacFL31 extended the shelf life and enhanced the sensory attributes of turkey meat samples stored at 4°C [15].

Despite that enterococci are considered as beneficial with technological properties; there has been increasing concern about the prevalence of virulence factors and antibiotic-resistance genes, which could compromise their foods application [26]. In this regard, enterocin-producing strains should be carefully assessed with regard to safety aspects before being used in food technology. Once their safety characterization and enterocin-mediated antagonism against foodborne pathogens and spoilage bacteria are confirmed, safe enterococci could be good candidates for potential use in bio-preservation.

In previous study, an* Enterococcus faecium *FL31 strain producing the enterocin BacFL31 was deeply studied for its antimicrobial activity and the probiotic properties and as well as safety aspects were characterized [15, 30, 31].

The present paper aimed to evaluate the potential bio preservative effect of BacFL31 alone or in combination with peel onion extract on ground beef meat during storage at 4°C. The microbial evaluation, the lipid and protein oxidation as well as sensory attributes were assessed. To our knowledge, combined addition of enterocin and plant extracts in meat products preservation has not been reported to date.

## 2. Materials and Methods

### 2.1. Bacterial Culture and Growth Conditions

The* E. faecium *FL31, enterocin BacFL31 producer strain, was characterized as described previously by Chakchouk-Mtibaa et al. (2014) [30]. This strain was grown in De Man, Rogosa and Sharp medium (MRS) broth at 37°C for 18 h [32].* L. monocytogenes* ATCC 19117 was used as target strain in the determination of bacteriocin and APOE activities and was cultured and counted on Brain Heart Infusion (BHI) medium. Serial dilutions were prepared, then, 0.1 mL volumes of each dilution were spread in BHI agar plates and incubated at 35°C for 48 h. Presumptive colonies of* L. monocytogenes* were counted and values were measured as CFU/mL on agar plates. The data represent results from three replicates.

### 2.2. Bacteriocin BacFL31 Preparation

A partially purified enterocin BacFL31 was recovered from a 900 mL of an 18h-old culture of* E. faecium *FL31 using a two purification step as described elsewhere [30]. To eliminate organic acids effect produced by this strain, the obtained active solution was neutralized at pH 6.5, concentrated to one-tenth of the original volume in a Rotavapor at 70°C, sterilized through a 0.45 *μ*m pore size filters (Millipore) and submitted to antimicrobial activity evaluation against* L. monocytogenes* ATCC 19117 using the agar well diffusion assay [33].

### 2.3. Aqueous Peel Onions Extract (APOE) Preparation

Onion peels extract was prepared with red onion peels provided by a local market in the region of Sfax - Tunisia. The collected onion peels were washed three times with distillated water and were shade-dried. The obtained dried onion peels was mechanically crushed with a food grinder (Moulinex Mixer Grinder LM2421). Then, the powders obtained were mixed with ultrapure water. The extract was filtered and then dried in a lyophilizer (Martin Christ, Alpha 1-2 LD plus Germany). The obtained extract was weighed and then mixed with water at a concentration of 20 mg/mL.

### 2.4. Quantitative Determination of Phenolic Compounds

#### 2.4.1. Total Polyphenols Content

Total polyphenols content of APOE was calculated according to the Folin-Ciocalteau method described by Waterman and Mole (1994) with some modifications [34]. Ten microliters of diluted extract solution was shaken for 5 min with 50 *μ*L of Folin-Ciocalteau reagent. Then 150 *μ*L of 20 % Na_2_CO_3_ was added. The obtained mixture was shaken once again for 1 min. Finally, the solution was brought up to 790 *μ*L by adding distilled water. After 2 hours, the absorbance at 760 nm was evaluated using a spectrophotometer. Gallic acid was used as a standard for the calibration curve. Total polyphenolics content (TPC) of the APOE was calculated according to the following equations:

(1) $$\begin{array}{c} Y=0.012\times x+0.017\;\;\left(\;R^{2}=0.997\;\right) \end{array}$$

TPC was expressed as *μ*g gallic acid equivalent per milligram of powder peel extract (*μ*g GA/mg) using the linear equation based on the calibration curve.

#### 2.4.2. Flavonoids Content

Flavonoids content in APOE was determined using the method of Quettier-Deleu et al. (2000) [35]. Briefly, 1 mL of AlCl_3_ was added to 1 mL diluted extract solution and vortexed and then incubated for 15 min in the dark. The absorbance at 430 nm was evaluated for the samples and the quercetin was used as standard for the calibration curve. Total flavonoids content (TFC) of the APOE was calculated according to the following equations:

(2) $$\begin{array}{c} y=0.051\times x+0.0003\;\;\left(\;R^{2}=0.999\;\right) \end{array}$$

TFC was expressed in *μ*g of quercetin equivalent per milligram of powder peel extract (*μ*g QE/mg).

#### 2.4.3. Tannins Concentration

The determination of the tannins was carried out according to the method of Julkunen-Titto (1985) [36]. 0.5 mL of APOE were mixed vigorously with three milliliters of 4 % vanillin in methanol. Immediately 1.5 mL of concentrated HCl was added to the mixture. The absorbance was read at 500 nm after 20 min at room temperature. Catechin was used as the standard. The tannin concentration (TAC) is expressed as catechin equivalents in mg per gram of extract (CE/g extract) and the content is obtained from the catechin calibration curve following the equation:

(3) $$\begin{array}{c} Y=0.5825\times x\;\;\left(\;R^{2}=0.918\;\right) \end{array}$$

### 2.5. Antioxidant Activity

Antioxidant activity of APOE was estimated by the measurement of the DPPH radical scavenging activity. This assay determines the scavenging effect of stable radical species according to the method of Kirby and Schmidt (1997) with slight modifications [37]. Briefly, the extract was diluted with ultrapure water at different concentrations (25; 50; 100, 200 and 400 *μ*g/mL). Then, 500 *μ*L of a DPPH radical solution (6 10^5^ M in HPLC grade methanol) was mixed with 500 *μ*L of samples. The mixture was incubated for 30 min in the dark at room temperature. Then, the absorbance of the resulting solution was read at 517 nm against a blank. The percentage of antiradical activity (% ArA) had been calculated as follows:

(4) %  ArA=Absorbance  of  Control−Absorbance  of  test  SampleAbsorbance  of  Control×100

The efficient concentration EC_50_ which represent the antioxidant amount necessary to decrease the initial DPPH concentration by 50 % was calculated from a calibration curve by linear regression. EC_50_ was expressed in terms of the concentration of sample extract in relation to the amount of initial DPPH (mg/mg DPPH). The antiradical power ARP was determined as the reciprocal value of the EC_50_ (mg/mg DPPH) following the equation:

(5) $$\begin{array}{c} ARP=\frac{100}{EC50} \end{array}$$

as described by kroyer (2004) [38].

### 2.6. Antibacterial Activity of the APOE and the Minimal Inhibitory Concentration (MIC) Determination

Minimal Inhibitory Concentration (MIC) of the APOE against* L. monocytogenes* ATCC 19117 was determined in BHI broth. The test was performed in sterile 96-well microplates with a final volume in each microplate well of 100 *μ*L. A stock solution of 20 mg/mL of APOE was two-fold serially diluted in LB medium. Ten *μ*L of* L. monocytogenes* ATCC 19117 cell suspension at 10^6^ CFU/mL were seeded in each microplate well. Then, plates were incubated overnight at 37°C. The MIC was defined as the lowest APOE concentration at which the microorganism does not demonstrate visible growth after incubation. Positive growth control wells consisted of bacterium only in their adequate medium. Cells suspension at the same concentration supplemented with ampicillin was used as control. Then, twenty five *μ*l of Thiazolyl Blue Tetrazolium Bromide (MTT) at 0.5 mg/mL were added to the wells and incubated at room temperature for 30 min. All experiments were performed in triplicate

### 2.7. Meat Samples Preparation

A fresh beef meat, purchased from a local supermarket (Sfax-Tunisia), was immediately transported to the laboratory at 4°C and was minced by grinding in a sterile grinder. Ground beef was divided into five equal lots: T_0_ (negative control: meat without any addition), T_1_ (positive control: meat added with 0.01% of the usual antioxidant BHT), T_2_ (meat supplemented with 200 AU/g of partially purified BacFL31), T_3_ (meat supplemented with active APOE at a concentration of 1 × MIC/g) and T_4_ (meat added with 200 AU/g of the partially purified BacFL31 combined with active APOE at a concentration of 1 × MIC/g).

These ingredients were homogenized in a blender (Moulinex Mixer Grinder LM2421) for 10 min, then packed in sterile plastic bags to produce three replicates and stored in a refrigerator at 4°C. Samples were withdrawn at 0, 3, 7, 10 14 days and analysed for: (i) microbial counts, (ii) physicochemical analysis consisting of metmyoglobin (MetMb), protein carbonyls, sulfhydryls groups, peroxide value (PV), thiobarbituric acid reactive substances (TBARS) and conjugated dienes (CD), and finally (iii) sensory attributes (color, texture, odour and overall acceptability).

### 2.8. Microbiological Analysis

Microbiological assays on meat samples were performed using international standard methods. Twenty five grams of meat were placed into a sterile stomacher bag and added to 225 mL of sterile buffered peptone water solution (0.1 g/100 mL). A 100 *μ*L of serial decimal dilutions were spread on the surface of agar plates. The International Organization for Standardization ISO 4833-2 [39], ISO 17410 [40] and ISO 21528-2 [41] were used respectively to enumerate aerobic plate counts (APC), aerobic psychrotrophic counts (PTC) and Enterobacteriaceae. Plates containing 25 - 250 colonies were selected and counted. The average number of CFU (colony forming units)/g was calculated and expressed as log_10_ CFU/g meat.

### 2.9. Physicochemical Analysis

#### 2.9.1. Lipid Oxidation

*(i) Peroxide Value (PV). *Peroxide values of samples were performed according to the method of Folch et al. (1957) [42]. Five grams of each sample were placed in a glass vial containing 50 mL of chloroform: methanol, 2:1 (*v/v*) and mixed in an orbital shaker at room temperature for 24 h. Subsequently, the homogenate was filtered using filter paper and washed with 15 mL of NaCl at 0.9 %. After a few seconds of vortexing, 10 mL of sample were collected from the bottom layer and evaporated under a stream of nitrogen gas, leaving the extracted lipids for PV analysis. The lipid sample was treated with 35 mL of a solvent mixture (acetic acid: chloroform, 3:2) and shaken thoroughly, then 0.5 mL of saturated potassium iodide solution was added. The mixture was kept in the dark for 5 min and 75 mL of distilled water were added followed by vigorous mixing. Soluble starch solution in phosphate buffer (2.5 mL at 1 %* w/v*) was used as an indicator. The peroxide value was determined by titration of the iodine liberated from potassium iodide using standardized 0.005 N sodium thiosulfate solutions. The PV was calculated by the following equation:

(6) $$\begin{array}{c} PV\left(\;mEq/Kg\;\right)=\frac{\left[\left(S-B\right)\times F\times 0.01\right]}{W}\times 1000 \end{array}$$

Where S is the volume (mL) of sodium thiosulfate required to titrate the sample; B is the volume (mL) of sodium thiosulfate required for the control; F is the calculated normality of the standardized sodium thiosulfate solution and W is the weight of the sample (g). The results are expressed as milli-equivalents of peroxide O_2_ per kg of meat.

*(ii) Thiobarbituric Acid Reactive Substance Value (TBARS). *Lipid oxidation was evaluated by thiobarbituric acid reactive substances (TBARS) according to the method described by Eymard et al. (2005) [43]. Two grams of sample were mixed with 100 *μ*L of butylated hydroxytoluene in ethanol at 1 g/L and 16 mL of trichloroacetic acid (TCA) at 50 g/L, then homogenized for 10 min and filtered. Two millilitres of filtrate (or 2 mL of TCA for blank) were added to 2 mL of thiobarbituric acid solution at 20 mol/L of concentration. The tube content was immediately vortexed and heated at 100°C for 15 min and rapidly cooled in ice. Absorbance was read against the blank at 508 (A_508  nm_), 532 (A_532  nm_) and 600 (A_600  nm_) with a spectrophotometer (Thermo Scientific/Genesys 20 Germany). The absorbance measured at the maximum (A_532  nm_) was corrected for the baseline drift as follows:

(7) A532  nm  corrected=A532  nm−A508  nm−A600  nm×600−532600/508−A600  nm

The results were expressed as mg of malonaldehyde equivalent per kg of sample (mg/kg) using the molar extinction coefficient of the MDA - TBA adduct at 532 nm (1.56 × 10^5^ M^−1^ cm^−1^) according to Buege and Aust (1978) [44]. The malonaldehyde equivalent was determined using the following equation:

(8) mg  MDAeq/kg=A  corrected×VTCA×2×MMDA×0.011.56×m

*(iii) Analysis of Conjugated Dienes. *One gram of each sample of beef meat was suspended in 10 mL of distilled water and homogenized. A 0.5 mL aliquot of this suspension was mixed with 5 mL of extracting solution: hexane: isopropanol at 3:1 (*v/v*) for 1 min, then centrifuged at 2000 ×* g* for 5 min. The absorbance of the supernatant was read at 233 nm. The concentration of conjugated dienes was calculated using the molar extinction coefficient of 25,200 M^−1^ cm^−1^ and the results were expressed as *μ*mole per mg of ground beef meat sample [45].

#### 2.9.2. Protein Oxidation

*(i) Metmyoglobin Analysis. *Metmyoglobin (MetMb) content was described by Krzywicki (1982) [46]. Briefly, 5 g of sample were placed into a 50 mL polypropylene centrifuge tube and homogenized with 25 mL of ice-cold phosphate buffer (40 mM at pH 6.80) for 1 min. The homogenized solution was kept at 4°C for 1 h and centrifuged at 4.500* × g* for 30 min at 4°C. The supernatant was filtered through 0.45 *μ*m pore size filters (Millipore), and absorbance was read at 572, 565, 545, and 525 nm using a spectrophotometer.

The MetMb percentages were then calculated based on those absorbance values using the following formula:

(9) MetMb%=−2.51A572nmA525nm+0.777A565nmA525nm+0.8A545nmA525nm+1.098×100

A refers to the corresponding absorbance.

*(ii) Determination of Carbonyls Contents. *The classical approach to the detection of protein carbonyl groups involves their reaction with 2,4-dinitrophenylhydrazine (DNPH) according to the method of Oliver et al. (1987) [47]. Two procedures were used for the determination of protein oxidation in meat sample: carbonyl content and protein quantification. One gram of ground beef sample was homogenized in 10 mL of 0.15 M KCl buffer for 60 sec at the speed of 20980* × g*. A 50 *μ*L of the resulting blend was transferred into an Eppendorf vial containing 1 mL of TCA at 10 % (*w/v*). Samples were centrifuged for 5 min at 2880* × g* and supernatant was removed. For carbonyl measurement, 1 mL of 2 M HCl containing 0.2 % 2,4- dinitrophenyl hydrazine (DNPH) and for proteins 1 mL of 2 M HCl was added to the Eppendorf vials. Samples were then incubated for 1 h at room temperature, with vortexing every 20 min. Following the incubation, 1 mL of 10 % TCA was added, vortexed and centrifuged again for 10 min at 2880* × g*. The supernatant was removed, and the pellet was washed twice with 1.5 mL of ethanol/ethyl acetate (1:1;* v/v*), shaken, and centrifuged for 5 min at 12000* × g*. After the complete removal of DNPH residues, the pellets were dried under N_2_ gas and dissolved in 1.5 mL of 6 M guanidine hydrochloride in 20 mM sodium phosphate buffer (final pH of 6.5), shaken, and centrifuged for 5 min at 4000* × g*.

*(iii) Determination of Sulfhydryl Groups. *Total free sulfhydryl groups (SH) content was determined by reacting with 5, 5′-dithiobis (2-nitrobenzoic acid: DTNB). According to Ellman (1959), a 0.5 g of meat sample was dissolved in 10 mL phosphate buffer (pH 7.2, 0.05 M) by shaking at room temperature for 1 hour [48]. Then, 1 mL of the homogenate was mixed with 9 mL phosphate containing 8 M urea, 0.6 M NaCl and 6 mM EDTA and the mixture was centrifuged for 20 min at 14000* × g *at 4°C. Three mL of supernatant were incubated with 1 mL DTNB reagent (0.01 M DTNB in 0.05 M sodium acetate) at 40°C for 15 min. The absorbance was measured at 420 nm. Control sample was run with 1.0 mL phosphate buffer without DTNB; reagent blank was run with water only. The sulfhydryl content was calculated based on sample absorbance using a molar extinction coefficient of 13600 M^−1^cm^−1^ and the results were expressed as mmol sulfhydryl per g of ground beef sample.

### 2.10. Sensory Evaluation

Sensory evaluation of ground beef meat was performed by a panel of 25 researchers at the Centre of Biotechnology of Sfax - Tunisia. Each panellist performs five different assays for meat samples. For each analysis (0, 3, 7, 10 and 14 days of storage at 4°C), each sample was evaluated in three sessions. The panellists scored the sensory color, texture, odour and overall acceptability attributes by using a 9-point hedonic scale (9 = like extremely, 8 = like very much, 7 = like moderately, 6 = like slightly, 5 = neither like nor dislike, 4 = dislike slightly, 3 = dislike moderately, 2 = dislike very much, 1 = dislike extremely). A score of 5 was taken as the lower limit of acceptability.

### 2.11. Statistical Analysis

The experiments were done in triplicate. The results are given as mean standard deviation (SD).

Student's t-test was used for comparison between two treatments at (*P* < 0.05).

A one-way analysis of variance (ANOVA) with two factors (treatments and storage time), was applied for each parameter by using SPSS 19 statistical package (SPSS Ltd., Woking, UK). Means and standard deviation were calculated and a probability level of* P* < 0.05 was used in testing the statistical significance of all experimental data. Tukey's post hoc test was used to determine significance of mean values for multiple comparison at (*P* < 0.05).

## 3. Results and Discussion

### 3.1. Total Phenolic, Total Flavonoid and Tannin Contents

Total phenolic (TPC), total flavonoid (TFC) and tannin (TAC) contents of APOE were determined and expressed in gallic acid equivalents (mg GAE/g), quercetin equivalents (mg QE/g) and (mg CE/g) respectively. As presented in Table 1, APOE had a high TPC of 140 mg GAE/g. Other studies reported similar TPC of 125 mg GAE/g for aqueous extract of peel onion at 165°C [49]. Same observations have been reported by Lee et al. (2014) when proving that the onion peel extracted by heated water for 3 h at 60°C contained 120.60 mg GAE/g [50].

The TFC of APOE, established by AlCl_3_ method, was about 35 mg QE/g (Table 1). Previous studies by Lee et al. (2014) showed that the hot water extract of onion peel contained 54.5 mg QE/mg of extract [50]. The quercetin compounds are major flavonoids in onions and are related to skin colors and disease in plant [50, 51]. Gorinstein et al. (2008) reported that red onions had twice higher quercetin levels than that of white onions [52]. By comparing different extraction methods, ethanol extraction showed greater concentrations of TPC and TFC, respectively, of 327.50 mg GAE/g and 183.95 mg QE/mg of extract [50].

The determination of TAC concentration reveals that the APOE contains 20.6 mg CE/g (Table 1). It should be noted that the phytochemical composition of onions is believed to vary according to species and cultivation technique. Among the species of onions, the red onion is known to be rich in polyphenols, flavonoids, flavonol, and tannin [53].

### 3.2. Evaluation of Antioxidant Activity

**Figure 1:**
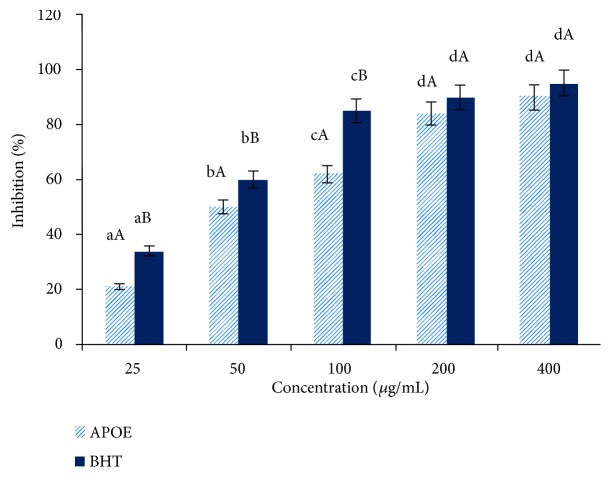
DPPH radical-scavenging activity of the APOE at different concentrations (25 - 400 *μ*g/mL) compared to the BHT. ±: Standard deviation of three replicates. A - B: A* t*-Student test was applied to determine the significant differences between treatments at* P *< 0.05; a - d: Tukey's post-hoc test was used to compare the significant differences at each concentration at* P* < 0.05.

DPPH is a stable free radical, which has been widely used as a tool for estimating free radical-scavenging activities of antioxidants substances [54]. Plants with radical scavenging property and antioxidant capacity are useful for medicinal applications and as food additive. So, in the present study the antioxidant capacity of APOE was evaluated using DPPH radical scavenging method by comparing with the activity of the BHT as a conventionally applied antioxidant. The DPPH radical-scavenging activity of the APOE with varying concentrations from 25 to 400 *μ*g/mL was determined and compared to the BHT activity (Figure 1). The antiradical activity assay of the APOE was dose-dependent. APOE at a concentration of 25 *μ*g/mL, showed the lowest radical activity in comparison with the free radical activity of the BHT, while at 400 *μ*g/mL, APOE revealed a very interesting DPPH activity in comparison with the BHT one (Figure 1).

In correlation with the high contents of TPC, TFC and TAC, APOE exerted effective radical scavenging activity with an efficient concentration EC_50_ of 0.05 mg/mL, respectively and 2.17 ± 0.10 mg/mg DPPH and an antiradical power (ARP) of 46 ± 1.51. In comparison of the study of Singh et al. (2009), ARP of aqueous fraction was 1.8 ± 0.3 [55]. The latter study demonstrated that ARPs of different fractions extracted by dichloromethane, diethyl ether, ethyl acetate, butanol and water were 1.2 ± 0.3, 4.9 ± 0.6, 75.3 ± 4.5, 13.4 ± 0.8 and 1.8 ± 0.3, respectively [55].

### 3.3. Antibacterial Activity of the APOE and MIC Determination

Minimal Inhibitory Concentration (MIC) of the APOE against* L. monocytogenes *has been determined and is equal to 1.56 ± 0.3 mg/mL as shown in Table 1.

### 3.4. Application of Enterocin BacFL31 Alone and in Combination with APOE during Conservation of Ground Beef Meat at 4°C

#### 3.4.1. Microbiological Characteristics

**Figure 2:**
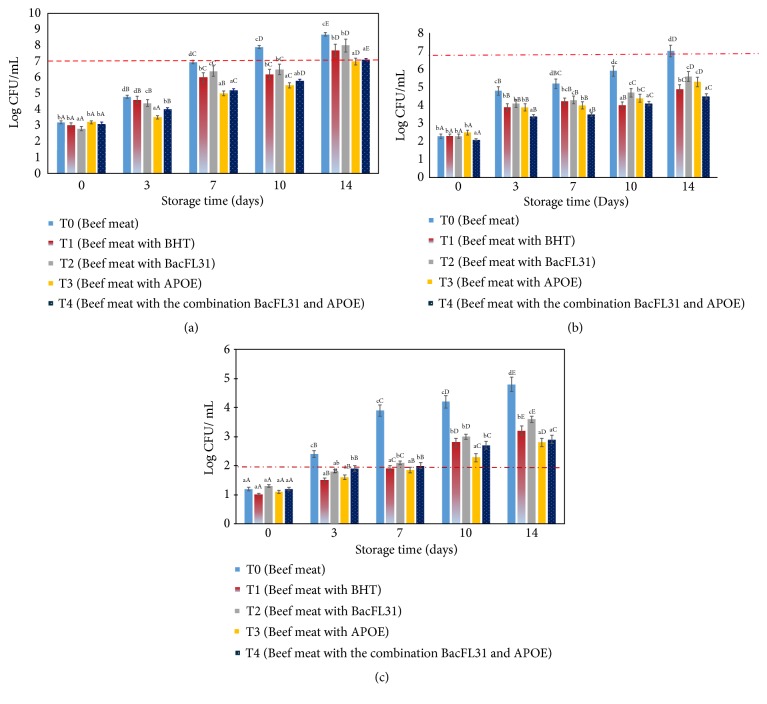
(a) Effect of the enterocin BacFL31 at 200AU/g, APOE at 1MIC/g, and the combination (BacFL31 + APOE) on the microbial load of APC of ground beef meat during storage at 4°C. ±: Standard deviation of three replicates. Values with a different letter (a - c) at the same storage day are significantly different (*P* < 0.05). Values with a different letter (A - D) of the same treatment are significantly different (*P *< 0.05) by using Tukey's post-hoc test. (b) Effect of the enterocin BacFL31 at 200AU/g, APOE at 1MIC/g, and the combination (BacFL31 + APOE) on the microbial load of PTC of ground beef meat during storage at 4°C. ±: Standard deviation of three replicates. Values with a different letter (a - c) at the same storage day are significantly different (*P* < 0.05). Values with a different letter (A - D) of the same treatment are significantly different (*P *< 0.05) by using Tukey's post-hoc test. (c) Effect of the enterocin BacFL31 at 200AU/g, APOE at 1MIC/g, and the combination (BacFL31 + APOE) on the microbial load of PTC of ground beef meat during storage at 4°C. ±: Standard deviation of three replicates. Values with a different letter (a - c) at the same storage day are significantly different (*P* < 0.05). Values with a different letter (A - D) of the same treatment are significantly different (*P *< 0.05) by using Tukey's post-hoc test.

The aerobic plate counts (APC), aerobic psychrotrophic counts (PTC) and Enterobacteriaceae counts of treated samples were significantly (*P* < 0.05) lower than those of control ones during storage (Figure 2).

APC of different samples was above 3.0 CFU/g (*P* > 0.05) at the beginning of storage period. After seven days of storage for the negative control sample (T_0_), APC value increased significantly (*P* < 0.05) with the increase of the storage time at 4°C and reached the minimal spoilage level at 7.0 log_10_ CFU/g [56]. During the storage period of 7 days, a gradual increase (*P *< 0.05) in the APC for all treated samples (T_1_, T_2_, T_3_ and T_4_) was observed and respectively reached 6.01, 6.4, 5.01 and 6.0 log_10_ CFU/g. For T_2_ sample, the minimal spoilage level was reached after 12 days of storage, while the APC counts recorded for T_3_ and T_4_ were noted to remain under the detection limits (7.0 log log_10_ CFU/g) until days 14 of storage. In fact, as illustrated in the Figure 2(a), T_3_ and T_4_ samples were most effective (*P* < 0.05) and could extend the shelf life storage 2 days than the meat treated with BacFL31 alone at 200 AU/g (T_2_).

As indicated in Figure 2(b), PTC of the treated samples by BacFL31 (T_2_), APOE (T_3_) and the combination Bac FL31 + APOE (T_4_) was lower (*P* < 0.05) than the untreated sample (T_0_). According to Speck (1984), a count of above 6.7 log CFU/g of psychrotrophic bacteria makes the product unsuitable for consumption for ground beef meat [57]. In our case, all treated samples never exceeded the maximal limit, while for the control samples (T_0_ and T_1_), 14 days are sufficient to attain this limit (Figure 2(b)). In a previous work, the PTC reduction on poultry meat has been reported by Chakchouk-Mtibaa et al. (2017) [15]. The authors proved that a treatment with 400 AU/g of enterocin BacFL31 could extend the shelf life of chicken breast to 15 days whereas the control samples started to deteriorate after eight days of storage. For T_3_ and T_4_ samples, the increase in APC and PTC was comparatively lower (*P* < 0.05) than control products which might be attributed to the presence of phenolic compounds [55].

For the negative control sample,* the Enterobacteriaceae *counts reached rapidly the detection limit which is 2 log CFU/g according to AFNOR V01-003 (2004) [56]. For the treatments T_1_, T_2_, T_3_ and T_4_, a significantly (*P* < 0.05) reduction of the* Enterobacteriaceae* count was observed and the standard limit was reached after seven days of storage at 4°C for all treatments. In previous work, 200 AU/g of BacFL31 was demonstrated to be able to reduce the growth of* Enterobacteriaceae *and extend the shelf life of raw ground turkey escalope to 10 days, which reached 14 days with concentrations of 400 AU/g [15]. Interestingly, in this current study, both the addition of 200 AU/g of BacFL31 (T_2_), APOE (T_3_) and their combination (T_4_) were able to reduce the growth of* Enterobacteriaceae *and extend the shelf life of raw ground beef meat to four days compared to the control samples (Figure 2(c)).

#### 3.4.2. Physicochemical Analyses

*(1) Development of Protein Oxidation Products*

*(i) Metmyoglobin (MetMb). *Meat color, depending on the chemical state of myoglobin, is an important factor that influences product acceptability by consumers. In fact, the undesirable discoloration of meat during preservation is largely due to myoglobin oxidation and the MetMb formation [58]. The changes of MetMb content in the ground beef meat during storage at 4°C are presented in Table 2. MetMb % increased rapidly in the first seven days of storage and reached values above 40.9% in the negative control sample (T_0_), whereas for treated samples (T1–T4) the MetMB percentage were ranged from 32.04 (T_4_) to 34.93 (T_1_). The treated samples T_2_ and T_3_ exceeded the limit of acceptability after ten days whereas, for the treated sample T_4_, the limit was attained after fourteen days of storage. It is worth noting that consumer rejection of meat products occurred at 40% of Met-Mb [58]. We can explain our results by the strong antioxidant properties of APOE due to its phenolic components [24, 55]. In fact, free radical scavengers could inhibit the formation of MetMb [59]

*(ii) Protein Carbonyls. *Carbonylation is generally recognized as one of the most remarkable chemical modifications in oxidized proteins [5]. The formation of carbonyl compound (aldehydes and ketones) in meat proteins principally derives from the oxidation of threonine, proline, arginine and lysine residues [5]. The BacFL31 and the APOE addition had very significant effect (*P* < 0.05) on the carbonyls formation (Table 2). During storage time, control negative sample had significantly (*P* < 0.05) higher values of protein carbonyls than the treated ones. At the first day of storage, no significant difference (*P* > 0.05) between the carbonyl contents values of the control sample and all treated samples: T_1_, T_2_, T_3_ and T_4_. The carbonyl level of control sample increased (*P* < 0.05) during storage reach a maximum values of 6,41 nmol/mg protein after seven days then decreased to 4.51 nmol/mg protein at the end of the storage period (Table 2).

For T_2_ sample, the amount of carbonyl groups reached its maximal value with a concentration of 5.45 nmol/mg protein lower (*P* < 0.05) than the control samples (T_0_ and T_1_). For T_3_ sample, the maximum value was reached at the same time with a concentration of 4.15 nmol/mg protein. The T_4_ sample was very efficient (*P* < 0.05) on preventing carbonyl formation. The maximum value of the carbonyl contents for the T_4_ treatment was approximately twice lower than the control sample T_0_ (Table 2). Similarly, the decrease in carbonyl groups under storage was reported for beef meat balls [60] and turkey meat sausage [14]. According to Estévez et al. (2011), the formation of protein carbonyls from particular amino acid side chains contribute to impair the conformation of myofibrillar proteins leading to denaturation and loss of functionality [61].

*(iii) Sulfhydryl Content. *Proteins may contain several actual or potential sulfhydryl groups. The measurement of thiol (sulfhydryl) content are an interesting way to evaluate free radical attack on proteins and to measure the degree of oxidative reactions in meat during refrigerated storage [61]. In fact, the determination of sulfhydryl groups concentration is an appropriate indicator of protein oxidation level [62]. During storage, concentration of sulfhydryl groups decreases (*P *< 0.05) with the progress of oxidative reaction. Treatments with BacFL31 (T_2_) and APOE (T_3_) were effective (*P* < 0.05) in the protection of SH groups against alteration by oxidation processes during refrigerated storage of the ground beef meat. As shown in Table 2, the maximum decrease was observed in control samples and the minimum decrease was observed in samples treated with the combination of the enterocin BacFL31 and the APOE (T_4_) with final sulfhydryl concentrations of 29.14 and 42.19 nmol/mg protein, respectively, at the end of storage. On the other hand, as seen in Table 2, no significant difference (*P* > 0.05) was observed between the meat added with APOE (T_3_) or added with the combination of APOE and BacFL31 (T_4_). These results indicated that the addition of plant extract (T_3_) inhibit the oxidation process and reduce the loss of sulfhydryl groups. Previous studies reported that the efficiency of plant extract was increased with the concentration of phenolic compounds [60, 62].

*(2) Development of Lipid Oxidation Products*

*(i) Peroxide Value (PV). *PV, an important characteristic of primary lipid oxidation, is the most used parameter for measuring the primary products of oxidative degradation in meat [14]. During the refrigerated storage at 4°C, as shown in the Table 2, treated samples had significantly (*P* < 0.05) lower PVs compared to the negative control sample (T_0_). For treated samples, the lowest (*P* < 0.05) was observed in the meat treated with the APOE (T_3_) alone or combined with the enterocin BacFL31 (T_4_). The latter was the most effective (*P* < 0.05) treatment to retard the primary auto-oxidation up to 14 days. These results are in accordance with the study of Shim et al. (2012) [24], who reported that raw samples containing 0.2 % peel onion extract exhibited lower PV than negative control and treated samples with ascorbic acid. The negative control sample reached the maximum value (14.2 meq peroxide/Kg of meat) after ten days of storage and then a rapid decrease (*P* < 0.05) was observed. This decrease in PV was related to hydroperoxide degradation and secondary lipid formation [60]. For the treated samples (T_2_, T_3_ and T_4_) and the positive control (T_1_) a slight increase was observed (*P* < 0.05) during storage. The maximum PVs were reached in samples T_2_, T_3_ and T_4_ and were respectively 10.75, 7.91, and 8.16 meq peroxide/Kg of meat. The slight significant (*P* < 0.05) increase observed indicated that the antibacterial effect of the enterocin BacFL31 and APOE delay the progression of initial oxidation step and the degradation of the formed peroxides. In accordance with our results, Mir et al. (2017) [63], reported that the addition of spices at level of 0.1 % caused decrement PV values in rista, a traditional meat product of India, compared to the control.

*(ii) TBARS. *TBARS is a reactive aldehyde produced by lipid peroxidation of meat polyunsaturated fatty acids [14]. TBARS values of ground beef meat are shown in Table 2. They were increased (*P* < 0.05) during storage in all samples. The TBARS values in the negative control sample (T_0_) were higher (*P* < 0.05) than treated samples. The control sample (T_0_) becomes unacceptable beyond 7 days of storage and a TBARS value of 2.12 mg MDA/kg of meat was recorded. According to Campo et al. (2006), an index of 2 mg MDA/kg of meat was considered the limiting threshold for the acceptability of oxidized beef meat [64]. For T_2_ sample, the limit of acceptability was reached after ten days of storage whereas the samples treated with the BHT (T1), APOE (T_3_), and the combination BacFL31+ APOE (T_4_) remained acceptable at the end of storage (Table 2).

These results showed that the enterocin BacFL31 and the aqueous peel onion extract addition can protect the ground beef meat against lipid oxidation and extend the shelf life of meat. The use of APOE was very effective against the development of oxidative rancidity in beef meat. The phenolic compounds present in the peel onion extract could be an efficient electron donor capable to react with free radicals during the oxidation reaction.

*(iii) Conjugated Dienes (CD). *The CD values in control and treated samples during refrigerated storage are presented in Table 2. CD analysis revealed that the treatments and storage period significantly (*P* < 0.05) affected the lipid oxidation of beef meat samples. During storage period, the CD value of the negative control sample was higher (*P* < 0.05) than the treated ones. As shown in Table 2, we noticed that the concentration of CD increased significantly (*P* < 0.05) for all treatments at the beginning then decreased until the end of storage. This decrease in CD values proved that the conjugated hydroperoxides are expected to be transformed to secondary products as the TBARS formation occurs [65]. These findings were in accordance with previous studies of turkey meat sausage treated with bacteriocin BacTN635 [14].

#### 3.4.3. Sensory Evaluation

The changes in attribute scores of sensory evaluation: color, texture, odor and overall acceptability of untreated (T_0_) and treated (T_1_, T_2_, T_3_ and T_4_) ground beef meat during the fourteen days of refrigerated storage are shown in Table 3. It should be noted that sensory attributes scores of meat samples untreated and treated with enterocin BacFL31 and APOE were assessed by the panellists with scores above the rejection limit set to 5. Furthermore, the addition of BacFL31 at 200 AU/g (T_2_), APOE (T_3_), and their combination (T_4_) and storage time have a significant effect (*P* < 0.05) on the sensory parameters of ground beef meat (Table 3). The negative control sample displayed the lowest score at day 14, demonstrating unacceptable odor, texture and color as well as a very low overall acceptability. Equally, at the end of the storage period (14 days), T_4_ sample showed the significant (*P* < 0.05) and highest color, texture, odor, and overall acceptability scores which were respectively 5.29 ± 0.14, 5.03 ± 0.23, 5.07 ± 0.15 and 4.80 ± 1.12 (Table 3). Whereas the negative control sample become unacceptable after 3 days of storage, the overall acceptability of ground beef meat treated with BacFL31 (T_2_) remains acceptable until 10 days of storage. The meat treated with the APOE (T_3_) and with combination (T_4_) remains acceptable for two more days than the meat treated with BacFL31 (T3).

## 4. Conclusion

In this study, we used two natural compounds in the preservation of the ground beef meat at 4°C during 14 days of storage. The bacteriocin BacFL31 at 200 AU/g from the safe strain* E. faecium* FL31 and the aqueous peel onion extract (APOE) at 1 MIC/g were added alone or in combination for meat biopreservation. The impact of the different treatments as regards microbiological, physico-chemical and sensory properties was evaluated. The use of the combination between bacteriocin and plant extract was significantly more effective than the use of each active compound alone. To the best of our knowledge, this is the first report using such combination and may provide novel solutions for improved meat safety. These findings provide interesting information for meat preservation, delaying lipid and protein oxidation and preventing the pathogens proliferation.

## Figures and Tables

**Table 1:** Total phenolic, total flavonoid and tannins contents, antioxidant and antibacterial activities of the aqueous extracts of onion peel.

Phytochemical contents				Antioxidant activity				Antibacterial activity
	TPC (mg GAE/g)	TFC (mg QE/g)	TAC (mg CE/g)		EC_50_ (mg/mL)	EC_50DPPH_ (mg/mg)	ARP	MIC (mg/mL)
APOE	140 ± 2.05	35 ± 0.5	20.6 ± 1.4	APOE	0.05 ± 0.00	2.17 ± 0.10	46 ± 1.51	1.56 ± 0.3
				BHT	0.033 ± 0.00	1.43 ± 0.07	69.69 ± 2.75	

TPC: total phenolic content. TFC: total flavonoid content. TAC: total tannin content. EC: efficient concentration. ARP: antiradical power. MIC: minimal inhibitory concentration.

±: standard deviation of three replicates.

**Table 2:** Effect of BacFL31, APOE, and their combination on MetMb (%), protein carbonyl (nmoles carbonyl/mg protein), sulfhydryls (nmoles sulfhydryl/mg protein), peroxide values (meq peroxide/Kg of meat), TBARS (mg MDA/kg meat) and the conjugated dienes (*µ*mol/mg of meat) of the ground beef meat during storage at 4°C.

Days of storage at 4°C					
	0	3	7	10	14
	*Protein oxidation products*				

*MetMb*					
T_0_	23.98 ± 0.02^aA^	35.13 ± 0.04^aB^	40.9 ± 0.12^aC^	51.33 ± 0.15^aD^	58.23 ± 0.22^aE^
T_1_	24.03 ± 0.11^aA^	31.15 ± 0.13^bB^	34.93 ± 0.1b^BC^	37.14 ± 2.06c^dC^	43.06 ± 2.51^bD^
T_2_	23.85 ± 0.10^aA^	31.12 ± 0.09^bA^	36.09 ± 0.12^cB^	42.94 ± 0.19^bC^	45.03 ± 2.22^bD^
T_3_	23.93 ± 0.17^aA^	27.1 2± 0.11^cB^	34.26 ± 0.10^dC^	39.54 ± 0.21^cD^	42.2 ± 0.11^bE^
T_4_	23.61 ± 0.11^aA^	26.34 ± 0.12^dB^	32.04 ± 0.11^eC^	35.14 ± 0.13^dD^	40.23 ± 0.09^bE^

*Carbonyls contents*					
T_0_	4.02 ± 0.9^aA^	5.11 ± 0.11^aAB^	6.41 ± 0.78^aB^	5.84 ± 0.74^aAB^	4.51 ± 0.69^aA^
T_1_	3.58 ± 0.11^aC^	4.3 ± 0.11^bA^	4.45 ± 0.08^bcA^	3.85 ± 0.11^bBC^	4.01 ± 0.15^aB^
T_2_	3.88 ± 0.11^aC^	4.5 ± 0.12^bB^	5.45 ± 0.18^abA^	4.1 ± 0.16^bC^	3.88 ± 0.13^aC^
T_3_	3.04 ± 0.16^aBC^	3.55 ± 0.10^cAB^	4.15 ± 0.48^cdA^	3.1 ± 0.17^bBC^	2.67 ± 0.10^bC^
T_4_	3.11 ± 0.33^aAB^	2.22 ± 0.20^dB^	3.23 ± 0.37^cA^	3.38 ± 0.41^bA^	2.29 ± 0.33^bB^

*Sulfhydryls groups*					
T_0_	45.88 ± 0.20^aA^	42.55 ± 0.19^bB^	39.14 ± 0.03^bC^	32.31 ± 1.11^dD^	29.14 ± 0.81^dE^
T_1_	45.45 ± 022^aA^	42.10 ± 2.10^bAB^	40.6 ± 0.08^abAB^	40.19 ± 0.81^bBC^	37.60 ± 0.22^bC^
T_2_	46.03 ± 0.93^aA^	42.45 ± 0.01^bAB^	39.07 ± 1.67^bAB^	36.68 ± 1.61^cAB^	33.55 ± 1.09^cC^
T_3_	45.53 ± 0.77^aA^	42.23 ± 1.01^bB^	43.07 ± 1.23^aC^	42.68 ± 1.22^abD^	40.55 ± 1.04^aE^
T_4_	46.07 ± 0.33^aA^	44.55 ± 1.06^aAB^	43.27 ± 1.55^aB^	43.08 ± 0.28^aB^	42.19 ± 1.09^aB^

	*Lipid oxidation products*				

*Peroxide values*					
T_0_	2.21 ± 0.51^aA^	6.32 ± 0.24^aB^	11.06 ± 0.41^aC^	14.22 ± 0.23^aD^	11.85 ± 0.82^aC^
T_1_	1.89 ± 0.57^aA^	4.74 ± 0.44^bB^	6.48 ± 0.11^bC^	7.9 ± 0.25^cD^	8.16 ± 0.64^abD^
T_2_	2.05 ± 0.45^aA^	5.05 ± 0.33^bB^	7.11 ± 0.25^bC^	9.48 ± 0.41^bD^	10.75 ± 0.32^abE^
T_3_	1.89 ± 0.17^aA^	3.95 ± 0.60^bB^	5.21 ± 0.11^cC^	7.58 ± 0.21^cD^	7.91 ± 0.45^abD^
T_4_	2.05 ± 0.22^aA^	4.11 ± 0.43^bB^	4.74 ± 0.31^cB^	7.58 ± 0.41^cC^	8.16 ± 0.32^cC^

*TBARS value*					
T_0_	0.48 ± 0.12^aA^	1.45 ± 0.07^aB^	2.12 ± 0.09^aC^	2.71 ± 0.11^aD^	2.98 ± 0.12^aD^
T_1_	0.43 ± 0.01^aA^	0.81 ± 0.14^abB^	1.19 ± 0.13^cdC^	1.58 ± 0.11^cD^	2.01 ± 0.12^cE^
T_2_	0.44 ± 0.12^aA^	1.09 ± 0.09^abB^	1.59 ± 0.12^bC^	2.01 ± 0.09^bD^	2.48 ± 0.08^bE^
T_3_	0.40 ± 0.07^aA^	0.8 ± 0.12^abB^	1.31 ± 0.13^bcC^	1.61 ± 0.11^cC^	2.08 ± 0.13^cD^
T_4_	0.48 ± 0.11^aA^	0.69 ± 0.12^bAB^	0. 9 ± 0.11^dB^	1.22 ± 0.13^dC^	1.88 ± 0.08^cD^

*CD*					
T_0_	0.717 ± 0.13^aA^	0.752 ± 0.24^aA^	0.685 ± 0.13^aA^	0.629 ± 0.29^aA^	0.626 ± 0.23^aA^
T_1_	0.667 ± 0.65^aA^	0.689 ± 0.01^aA^	0.642 ± 0.09^aA^	0.616 ± 0.36^aA^	0.585 ± 0.21^aA^
T_2_	0.663 ± 0.33^aA^	0.681 ± 0.10^aA^	0.655 ± 0.14^aA^	0.613 ± 0.35^aA^	0.555 ± 0.23^aA^
T_3_	0.633 ± 0.14^aA^	0.686 ± 0.022^aA^	0.613 ± 0.11^aA^	0.525 ± 0.10^aA^	0.435 ± 0.13^aA^
T_4_	0.64 3± 0.22^aA^	0.681 ± 0.55^aA^	0.603 ± 0.33^aA^	0.505 ± 0.21^aA^	0.412 ± 0.22^bB^

±: standard deviation of three replicates. Values with a different letter (a - c) within a row of the same storage day of each treatment are significantly different (*P* < 0.05). Values with a different letter (A - E) within a column of the same treatment are significantly different (*P* < 0.05) by using Tukey's post-hoc test.

**Table 3:** Effect of BacFL31 and APOE and their combination on color, texture, odor, and overall acceptability of ground beef meat during storage at 4°C.

Days of storage at 4°C					
	0	3	7	10	14
*Color*					
T_0_	7.18 ± 0.24^cD^	5.68 ± 0.14^bC^	3.43 ± 0.31^aB^	2.92 ± 0.10^aA^	2.71± 0.14^aA^
T_1_	6.43 ± 0.17^abD^	6.23 ± 0.16^aC^	5.18 ± 0.15^bB^	5.11 ± 0.21^bB^	4. 91± 0.23^aB^
T_2_	6.91 ± 0.44^bD^	6.81 ± 0.33^cD^	6.11 ± 0.09^dC^	5.75 ± 0.32^dB^	5.19 ± 0.25^dA^
T_3_	6.51 ± 0.23^aE^	6.35 ± 0.21^aD^	5.93 ± 0.12^cC^	5.5 5± 0.29^cB^	5.0 6± 0.23^cA^
T_4_	6.71 ± 0.22^abE^	6.31 ± 0.25^aD^	6.11 ± 0.12^dC^	5.79 ± 0.13^eB^	5.29 ± 0.14^dA^

*Texture*					
T_0_	7.06 ± 0.17^cD^	5.13 ± 0.27^aC^	3.63 ± 0.12^aB^	3.41 ± 0.15^aB^	2.1 ± 0.15^aA^
T_1_	6.81 ± 0.42^bE^	6.62 ± 0.55^dD^	5.44 ± 0.33^bC^	5.14 ± 0.22^bcB^	4.51± 0.20^bA^
T_2_	6.55 ± 0.12^aE^	6.25 ± 0.14^cD^	6.00 ± 0.12^dC^	5.19 ± 0.22^cB^	4.81± 0.25^dA^
T_3_	6.56 ± 0.14^aE^	6.06 ± 0.13^bD^	5.44 ± 0.12^bC^	5.09 ± 0.25^bB^	4.73 ± 0.11^dA^
T_4_	6.88 ± 0.13^bD^	6.18 ± 0.15^cC^	5.81 ± 0.34^cB^	5.13 ± 0.21^bcA^	5.03 ± 0.23^cA^

*Odor*					
T_0_	7.06 ± 0.18^cD^	4.55 ± 0.15^aC^	3.25 ± 0.16^aB^	2.12 ± 0.11^aA^	2.03 ± 0.13^aA^
T_1_	6.73 ± 0.10^aE^	6.12 ± 0.10^dD^	5.17 ± 0.17^bC^	3.88 ± 0.39^bB^	3.31 ± 0.29^bA^
T_2_	6.63 ± 0.15^aE^	5.93 ± 0.16^cD^	5.24 ± 0.13^bcC^	4.23 ± 0.29^cB^	3.66 ± 0.17^cA^
T_3_	6.78 ± 0.11^abD^	5.64 ± 1.24^bC^	5.30 ± 0.11^cB^	5.10 ± 0.10^dA^	5.01± 0.12^dA^
T_4_	6.81 ± 0.17^bD^	5.80 ± 0.27^cC^	5.29 ± 0.13^cB^	5.22 ± 0.21^dB^	5.0 7± 0.15^dA^

*Overall acceptability*					
T_0_	6.93 ± 0.13^cD^	4.87 ± 0.18^aC^	4.15 ± 0.14^aB^	3.9 ± 0.42^aB^	2.93 ± 0.32^aA^
T_1_	6.75 ± 0.29^bE^	6.2 5± 0.18^cD^	5.53 ± 0.12^cC^	5.31± 0.10^cB^	3.88 ± 0.10^bA^
T_2_	6.77 ± 0.05^bE^	6.0 6 ± 0.04^bD^	5.31± 0.13^bC^	5.04 ± 0.16^bB^	4.77 ± 0.26^cA^
T_3_	6.52 ± 0.19^aE^	5.93 ± 0.11^bD^	5.33 ± 0.17^bC^	5.14 ± 0.02^bcB^	4.80 ± 1.12^cA^
T_4_	6.96 ± 0.24^cE^	6.27 ± 0.20^cD^	5.92 ± 1.11^dC^	5.12 ± 0.27^bB^	4.85 ± 0.23^cA^

±: standard deviation of three replicates. Values with different letter (a - c) within a row of the same storage day of each treatment are significantly different (*P* < 0.05). Values with a different letter (A - E) within a column of the same treatment are significantly different (*P* < 0.05) by using Tukey's post-hoc test.

## Data Availability

The safety Enterococcus faecium FL31 strain, the enterocin BacFL31, the aqueous peel onion and their results of the treated ground beef meat data used to support the findings of this study are included within the article.

## References

[1] Bhunia A. K. (2018). Foodborne microbial pathogens: mechanisms and pathogenesis. *in Food science Text*.

[2] Aziz M., Karboune S. (2018). Natural antimicrobial/antioxidant agents in meat and poultry products as well as fruits and vegetables: a review. *Critical Reviews in Food Science and Nutrition*.

[3] Pateiro M., Barba F. J., Domínguez R. (2018). Essential oils as natural additives to prevent oxidation reactions in meat and meat products: a review. *Food Research International*.

[4] Khan M. A., Ali S., Yang H. (2019). Improvement of color, texture and food safety of ready-to-eat high pressure-heat treated duck breast. *Food Chemistry*.

[5] Silva F. A. P., Estévez M., Ferreira V. C. S. (2018). Protein and lipid oxidations in jerky chicken and consequences on sensory quality. *LWT- Food Science and Technology*.

[6] Mariutti L. R. B., Bragagnolo N. (2017). Influence of salt on lipid oxidation in meat and seafood products: a review. *Food Research International*.

[7] Hajji H., Joy M., Ripoll G. (2016). Meat physicochemical properties, fatty acid profile, lipid oxidation and sensory characteristics from three North African lamb breeds, as influenced by concentrate or pasture finishing diets. *Journal of Food Composition and Analysis*.

[8] Lorido L., Ventanas S., Akcan T., Estévez M. (2016). Effect of protein oxidation on the impaired quality of dry-cured loins produced from frozen pork meat. *Food Chemistry*.

[9] Berardo A., Claeys E., Vossen E., Leroy F., De Smet S. (2015). Protein oxidation affects proteolysis in a meat model system. *Meat Science*.

[10] Ferreira V. C. S., Morcuende D., Madruga M. S., Silva F. A. P., Estévez M. (2018). Role of protein oxidation in the nutritional loss and texture changes in ready-to-eat chicken patties. *International Journal of Food Science & Technology*.

[11] Echegaray N., Gómez B., Barba F. J. (2018). Chestnuts and by-products as source of natural antioxidants in meat and meat products: a review. *Trends in Food Science & Technology*.

[12] Nikoo M., Regenstein J. M., Ahmadi G. H. (2018). Antioxidant and antimicrobial activities of (-)-epigallocatechin-3-gallate (EGCG) and its potential to preserve the quality and safety of foods. *Comprehensive Reviews in Food Science and Food Safety*.

[13] Smaoui S., Hsouna A. B., Lahmar A. (2016). Bio-preservative effect of the essential oil of the endemic Mentha piperita used alone and in combination with BacTN635 in stored minced beef meat. *Meat Science*.

[14] Smaoui S., Ennouri K., Chakchouk-Mtibaa A. (2017). Relationships Between Textural Modifications, Lipid and Protein Oxidation and Sensory Attributes of Refrigerated Turkey Meat Sausage Treated with Bacteriocin BacTN635. *Food and Bioprocess Technology*.

[15] Chakchouk-Mtibaa A., Smaoui S., Ktari N. (2017). Biopreservative efficacy of bacteriocin BacFL31 in raw ground Turkey meat in terms of microbiological, physicochemical, and sensory qualities. *Biocontrol Science*.

[16] Shah M. A., Bosco S. J. D., Mir S. A. (2014). Plant extracts as natural antioxidants in meat and meat products. *Meat Science*.

[17] Kanatt S. R., Tari S., Chawla S. P. (2018). Encapsulation of extract prepared from irradiated onion scales in alginate beads: a potential functional food ingredient. *Journal of Food Measurement and Characterization*.

[18] Manohar C. M., Xue J., Murayyan A., Neethirajan S., Shi J. (2017). Antioxidant activity of polyphenols from Ontario grown onion varieties using pressurized low polarity water technology. *Journal of Functional Foods*.

[19] Khan S. A., Jameel M., Kanwal S., Shahid S. Medicinal importance of Allium species: a current review.

[20] Tang X., Cronin D. A. (2007). The effects of brined onion extracts on lipid oxidation and sensory quality in refrigerated cooked turkey breast rolls during storage. *Food Chemistry*.

[21] Cao Y., Gu W., Zhang J. (2013). Effects of chitosan, aqueous extract of ginger, onion and garlic on quality and shelf life of stewed-pork during refrigerated storage. *Food Chemistry*.

[22] Kato T., Michikoshi K., Minowa Y.-I., Maeda Y., Kikugawa K. (1998). Mutagenicity of cooked hamburger is reduced by addition of onion to ground beef. *Mutation Research/Genetic Toxicology and Environmental Mutagenesis*.

[23] Lee J., Kim D.-H., Shin H.-S. (2008). Influence of onion (Allium cepa L.) on genotoxic heterocyclic amine formation and overall mutagenicity in fried hamburger patty. *Korean Journal of Food Science and Technology*.

[24] Shim S.-Y., Choi Y.-S., Kim H.-Y. (2012). Antioxidative properties of onion peel extracts against lipid oxidation in raw ground pork. *Food Science and Biotechnology*.

[25] Smaoui S., Elleuch L., Ben Salah R. (2014). Efficient role of BacTN635 on the safety properties, sensory attributes, and texture profile of raw minced meat beef and chicken breast. *Food Additives and Contaminants - Part A Chemistry, Analysis, Control, Exposure and Risk Assessment*.

[26] Rehaiem A., Belgacem Z. B., Edalatian M. R. (2014). Assessment of potential probiotic properties and multiple bacteriocin encoding-genes of the technological performing strain *Enterococcus faecium* MMRA. *Food Control*.

[27] Ananou S., Rivera S., Madrid M. I., Maqueda M., Martínez-Bueno M., Valdivia E. (2018). Application of enterocin AS-48 as biopreservative in eggs and egg fractions: synergism through lysozyme. *Food Science and Technology*.

[28] Blázquez I. O., Burgos M. J. G., Pérez-Pulido R., Gálvez A., Lucas R. (2018). Treatment with high-hydrostatic pressure, activated film packaging with thymol plus enterocin AS-48, and its combination modify the bacterial communities of refrigerated sea bream (Sparus aurata) fillets. *Frontiers in Microbiology*.

[29] Huang Y., Ye K., Yu K., Wang K., Zhou G. (2016). The potential influence of two *Enterococcus faecium* on the growth of *Listeria monocytogenes*. *Food Control*.

[30] Chakchouk-Mtibaa A., Elleuch L., Smaoui S. (2014). An antilisterial bacteriocin BacFL31 produced by *Enterococcus faecium* FL31 with a novel structure containing hydroxyproline residues. *Anaerobe*.

[31] Chakchouk-Mtibaa A., Sellem I., Kamoun Y., Smaoui S., Karray-Rebai I., Mellouli L. (2018). Safety aspect of *Enterococcus faecium* FL31 strain and antibacterial mechanism of its hydroxylated bacteriocin BacFL31 against *Listeria monocytogenes*. *BioMed Research International*.

[32] de Man J. C., Rogosa M., Sharpe M. E. (1960). A medium for the cultivation of *Lactobacilli*. *Journal of Applied Bacteriology*.

[33] Tagg J. R., McGiven A. R. (1971). Assay system for bacteriocins. *Journal of Applied Microbiology*.

[34] Mole S., Waterman S. (1994). Analysis of phenolic plant metabolites. *In Blackwell Scientific Publications*.

[35] Quettier-Deleu C., Gressier B., Vasseur J. (2000). Phenolic compounds and antioxidant activities of buckwheat (Fagopyrum esculentum Moench) hulls and flour. *Journal of Ethnopharmacology*.

[36] Julkunen-Tiitto R. (1985). Phenolic constituents in the leaves of Northern willows: methods for the analysis of certain phenolics. *Journal of Agricultural and Food Chemistry*.

[37] Kirby A. J., Schmidt R. J. (1997). The antioxidant activity of Chinese herbs for eczema and of placebo herbs - I. *Journal of Ethnopharmacology*.

[38] Kroyer G. T. (2004). Red clover extract as antioxidant active and functional food ingredient innovative. *Food Science & Emerging Technologies*.

[39] ISO 4833-2 International organization for standardization. Microbiology of the food chain - horizontal method for the enumeration of microorganisms.

[40] ISO 17410 Microbiology of food and animal feeding stuffs — horizontal method for the enumeration of psychrotropic microorganisms.

[41] ISO 21528-2 International organization for standardization. microbiology of food and animal feeding stuffs - horizontal methods for the detection and enumeration of enterobacteriaceae. Part 2: colony-count method.

[42] Folch J., Lees M., Sloane Stanley G. H. (1957). A simple method for the isolation and purification of total lipides from animal tissues. *The Journal of Biological Chemistry*.

[43] Eymard S., Carcouët E., Rochet M.-J., Dumay J., Chopin C., Genot C. (2005). Development of lipid oxidation during manufacturing of horse mackerel surimi. *Journal of the Science of Food and Agriculture*.

[44] Buege J. A., Aust S. D. (1978). Microsomal lipid peroxidation. *Methods in Enzymology*.

[45] Srinivasan S., Xiong Y. L., Decker E. A. (1996). Inhibition of Protein and Lipid Oxidation in Beef Heart Surimi-like Material by Antioxidants and Combinations of pH, NaCl, and Buffer Type in the Washing Media. *Journal of Agricultural and Food Chemistry*.

[46] Krzywicki K. (1982). The determination of haem pigments in meat. *Meat Science*.

[47] Oliver C. N., Ahn B.-W., Moerman E. J., Goldstein S., Stadtman E. R. (1987). Age-related changes in oxidized proteins. *The Journal of Biological Chemistry*.

[48] Ellman G. L. (1959). Tissue sulfhydryl groups. *Archives of Biochemistry and Biophysics*.

[49] Lee K. A., Kim K.-T., Nah S.-Y., Chung M.-S., Cho S. W., Paik H.-D. (2011). Antimicrobial and antioxidative effects of onion peel extracted by the subcritical water. *Food Science and Biotechnology*.

[50] Lee K. A., Kim K.-T., Kim H. J. (2014). Antioxidant activities of onion (Allium cepa L.) peel extracts produced by ethanol, hot water, and subcritical water extraction. *Food Science and Biotechnology*.

[51] Lee S. G., Parks J. S., Kang H. W. (2017). Quercetin, a functional compound of onion peel, remodels white adipocytes to brown-like adipocytes. *The Journal of Nutritional Biochemistry*.

[52] Lee B., Jung J.-H., Kim H.-S. (2012). Assessment of red onion on antioxidant activity in rat. *Food and Chemical Toxicology*.

[53] Gorinstein S., Leontowicz H., Leontowicz M. (2010). The influence of raw and processed garlic and onions on plasma classical and non-classical atherosclerosis indices: investigations in vitro and in vivo. *Phytotherapy Research: An International Journal Devoted to Pharmacological and Toxicological Evaluation of Natural Product Derivatives*.

[54] Martins N., Barros L., Dueñas M., Santos-Buelga C., Ferreira I. C. F. R. (2015). Characterization of phenolic compounds and antioxidant properties of Glycyrrhiza glabra L. rhizomes and roots. *RSC Advances*.

[55] Singh B. N., Singh B. R., Singh R. L. (2009). Polyphenolics from various extracts/fractions of red onion (Allium cepa) peel with potent antioxidant and antimutagenic activities. *Food and Chemical Toxicology*.

[56] AFNOR

[57] M. L. Speck

[58] Sarıçoban C., Yilmaz M. T. (2014). Effect of thyme/cumin essential oils and butylated hydroxyl anisole/butylated hydroxyl toluene on physicochemical properties and oxidative/microbial stability of chicken patties. *Poultry Science*.

[59] Huang B., He J., Ban X., Zeng H., Yao X., Wang Y. (2011). Antioxidant activity of bovine and porcine meat treated with extracts from edible lotus (Nelumbo nucifera) rhizome knot and leaf. *Meat Science*.

[60] Turgut S. S., Soyer A., Işıkçı F. (2016). Effect of pomegranate peel extract on lipid and protein oxidation in beef meatballs during refrigerated storage. *Meat Science*.

[61] Estévez M. (2011). Protein carbonyls in meat systems: a review. *Meat Science*.

[62] Lara M. S., Gutierrez J. I., Timón M., Andrés A. I. (2011). Evaluation of two natural extracts (Rosmarinus officinalis L. and Melissa officinalis L.) as antioxidants in cooked pork patties packed in MAP. *Meat Science*.

[63] Ahmad Mir S., Ahmad Masoodi F., Raja J. (2017). Influence of natural antioxidants on microbial load, lipid oxidation and sensorial quality of rista—A traditional meat product of India. *Food Bioscience*.

[64] Campo M. M., Nute G. R., Hughes S. I., Enser M., Wood J. D., Richardson R. I. (2006). Flavour perception of oxidation in beef. *Meat Science*.

[65] Cagdas E., Kumcuoglu S. (2015). Effect of grape seed powder on oxidative stability of precooked chicken nuggets during frozen storage. *Journal of Food Science and Technology*.

